# Study on the damage and failure behavior of coal exposed to acid corrosion under impact load

**DOI:** 10.1371/journal.pone.0338828

**Published:** 2025-12-18

**Authors:** Zhaoying Chen, Wenmei Han, Xiang Zheng, Hongtai Liu

**Affiliations:** 1 State Key Laboratory of Coal and CBM Co-mining, Jincheng, China; 2 Department of Engineering Mechanics, North University of China, Taiyuan, China; China University of Mining and Technology, CHINA

## Abstract

The depth of coal mining is continuously increasing. In the process of deep underground coal mining, the hydrological conditions of coal seams are complex, with some coal seams subjected to corrosion by acidic water. Acidic water-rock chemical interactions significantly degrade the mechanical properties of coal rocks. Concurrently, dynamic loads from frequent blasting and mining-induced rockbursts pose severe threats to mining safety. Investigating the dynamic properties of coal exposed to acidic water environments is crucial for ensuring safe coal mining operations. This study investigated anthracite coal as the research subject. Dynamic Brazilian splitting tests were conducted using a Split Hopkinson Pressure Bar (SHPB) apparatus to evaluate the mechanical behaviour of coal samples. A three-dimensional SHPB numerical model was constructed using the continuous-discrete coupling method and Flat Joint Model(FJM). The damage mechanism of coal under the action of acidic solutions was analyzed from the perspectives of dynamic mechanical response, damage evolution, and failure modes. The results indicate that the dynamic tensile strength of coal samples after exposure to acidic solutions is lower than that of raw coal samples, with strength decreasing in order: raw coal > pH 4 > pH 2. Moreover, with increasing impact velocity or loading rate, the dynamic tensile strength, degree of fragmentation, and dissipated energy of three types of samples increase linearly. In the dynamic stress–time response curve, during the initial stress rise phase, the model undergoes approximately linear elastic deformation with only a small number of microcracks forming. During unloading, the number of cracks continues to increase but at a reduced rate. Under the same impact velocities, the total number of cracks formed upon failure follows the order: pH2 > pH4 > raw coal.

## 1. Introduction

With the increasing depth and duration of coal mining operations, the hydrogeological conditions of mines in China have evolved from relatively simple to predominantly complex systems. Mine water is primarily accumulated water from abandoned mine areas, caused by water seeping into mine shafts, working faces, or tunnels from aquifers, surface water bodies, water-bearing fracture zones, karst caves, collapse columns, or tectonic fracture zones. The water, containing impurities, reacts with pyrite and other sulfur-containing minerals in the coal seam, resulting in a significant increase in acidic ions in the mine water [1]. Acidic water primarily reacts chemically with coal, causing dissolution, hydrolysis, ion exchange, and redox reactions [2]. These effects alter the microstructure of coal, leading to degradation of its macroscopic mechanical properties. During underground coal mining and excavation processes, the coal-rock mass in tunnels inevitably undergoes frequent blasting and mining-induced ground pressure, as well as other intense dynamic loads. Dynamic disasters of coal and methane outbursts also occur from time to time, all of which pose serious threats to coal mining safety [3,4].

A significant proportion of mine water now originates from accumulated water in goaf areas and typically exhibits acidic characteristics. Consequently, it has become critically important to investigate the stability of coal and rock masses under the influence of acidic mine water, particularly in relation to dynamic phenomena such as rockbursts. Dynamic impact failure in coal and rock masses is an inherently complex fracturing process, rendering the characterisation of their dynamic mechanical behaviour particularly challenging. Under dynamic loading, key mechanical properties—such as dynamic strength, failure strain, dynamic elastic modulus, and failure time—differ markedly from those observed under purely static loading conditions [5,6]. Yu et al. [7], using a Split Hopkinson Pressure Bar (SHPB) system, investigated the dynamic fracture toughness of limestone subjected to chemical solutions. The study demonstrated that with increasing degrees of chemical damage, fracture modes became more complex, the fractal dimension of fracture surfaces increased, and both dynamic fracture toughness and fracture energy decreased. Tao et al. [8] conducted dynamic compression tests, thin-section analysis, and SEM examinations on marble treated with acidic solutions. Their findings revealed that the acidic environment compromised intergranular cohesion and microstructure, leading to reductions in dynamic uniaxial compressive strength, elastic modulus, P-wave velocity, and acoustic impedance, while increasing the specific surface energy as the pH decreased. Yang et al. [9] investigated the CO_2_-H_2_O-rock interactions of granite under different reaction temperatures and reaction times. Dissolution enhanced connectivity by expanding the effective pore volume, increasing the mesoporous and microporous surface area, and reducing the average pore diameter. Huan et al. [10] performed one-dimensional impact tests on sandstone exposed to acidic solutions using SHPB apparatus, and proposed a comprehensive analytical framework for assessing chemically induced damage. Xue et al. [11] conducted impact experiments on chemically treated white sandstone using a combined static–dynamic loading device. Their results indicated that increased porosity and macropore proportions led to diminished mechanical performance under both static and dynamic conditions, with a nonlinear relationship observed between mechanical properties and chemical damage variables. Wang et al. [12] employed the Particle Flow Code (PFC) to simulate hydraulic fracturing processes in coal seams. The numerically derived fracture pressures and radii exhibited good agreement with field measurements, thereby validating the simulation’s reliability. Liu et al. [13] improved the conventional fluid–solid coupling algorithm in PFC, capturing the preferential flow behaviour of fractures after rock failure. Peng et al. [14] constructed a structurally complex joint network model using PFC to investigate the failure mechanisms of jointed rock slope systems. Wang et al. [15] developed a PFC2D model for Brazilian splitting tests on coal samples based on moment tensor theory, revealing the spatiotemporal characteristics and rupture strength of acoustic emission (AE) events during the failure process. Gao et al. [16] explored AE characteristics of coal under cyclic loading through discrete element simulations, introducing the concept of acoustic emission rate as a potential indicator of coal instability. Sun [17] and Xu et al. [18] conducted PFC2D-based simulations of Brazilian splitting tests on high-temperature-treated sandstone, elucidating the effects of elevated temperatures on crack propagation. Bai et al. [19] established a discrete element model of a Brazilian disc with double pre-existing flaws, demonstrating that failure was driven primarily by the initiation, propagation, and coalescence of microcracks in stress concentration zones. Han et al. [20] developed a two-dimensional SHPB discrete element model to examine the effects of flaw geometry and strain rate on the dynamic behaviour of sandstone. Tang et al. [21] simulated the SHPB splitting system using a continuum – discontinuum coupling approach, analyzing the influence of loading rate and bedding angle on the dynamic response of shale.

Previous research on the effects of acidic solutions on the physic-mechanical properties of rock has predominantly focused on materials such as sandstone, marble, granite, and limestone, which are commonly encountered in civil engineering. In contrast, relatively limited attention has been given to the dynamic mechanical behaviour of coal in the field of coal mining engineering. The dynamic response characteristics of coal rock serves as the basis for tunnel blasting excavation, support engineering design, and stress wave propagation patterns. Studying the dynamic properties of coal rock in acidic water environment is of great significance for ensuring safe mining of coal. Given that the tensile strength of coal is significantly lower than its compressive strength, failure in coal bodies typically occurs through tensile fracturing.

This study investigated the tensile mechanical properties of coal subjected to acidic solutions. A combined approach involving both laboratory experiments and numerical simulations was employed to analyze the dynamic tensile strength and damage mechanism of coal under acidic conditions.

## 2. Experimental

### 2.1. Preparation of coal specimens

**Fig 1:**
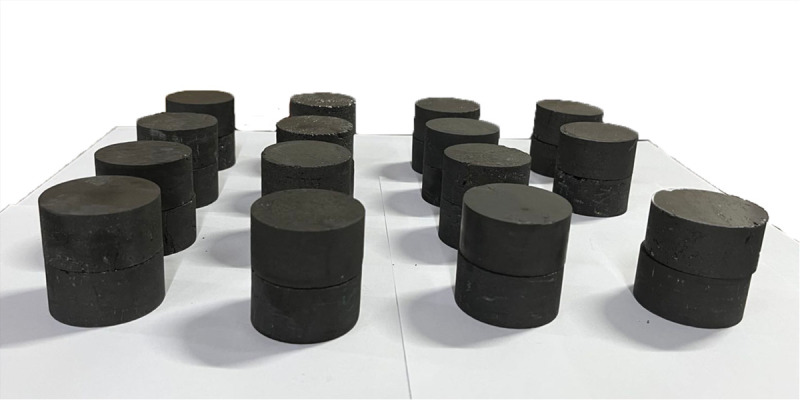
Brazilian Disc Specimens.

The coal samples used in this study were collected from the 3101 working face coal of the Yangcheng mine in Shanxi Province, China, which is situated within the Qinshui coalfield. They are classified as anthracite. A total of 30 standard Brazilian disc specimens were prepared, each with a diameter of 50 mm and a height of 25 mm. The top and bottom surfaces of each specimen were carefully ground to ensure they were as smooth and flat as possible, maintaining parallelism between the two ends. The specimens were divided equally into three groups, with 10 samples in each group, as shown in the Fig 1 below.

Duo to the influence of geological movements and anthropogenic activities, mine water has a complex composition, including acidic ions such as SO_4_^2-^, Cl^-^, and HCO_3_^-^.The pH of acid mine water may range from 2 to 8 [22]. Mixed acidic solutions with pH values of 4 and 2 were prepared using distilled water and standard solutions of 1 mol/L HCl and NaHSO_4_. Specimens R-1 to R-10 represent raw coal samples, while specimens P4-1 to P4-10 and P2-1 to P2-10 correspond to samples treated with pH = 4 and pH = 2 acidic solutions, respectively. All treated specimens were immersed in the respective acidic solutions for 24 hours, followed by drying at 50 °C for 12 hours according to the practice widely used for acid corrosion studies [23,24].

### 2.2. Experimental apparatus and principle

**Fig 2:**
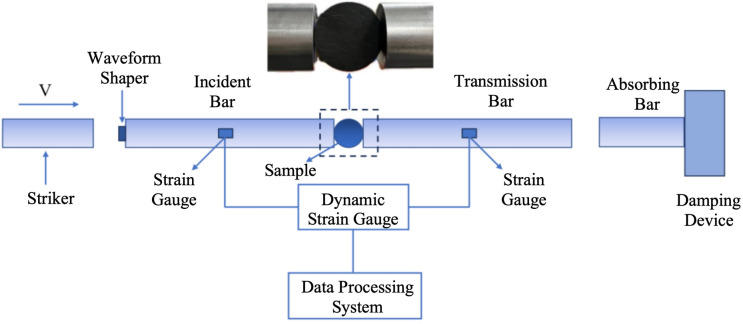
SHPB schematic.

Dynamic Brazilian splitting tests on coal specimens were conducted using a SHPB apparatus. The system utilized a 50 mm diameter separated Hopkinson pressure bar setup, comprising loading and driving mechanisms, pressure bars, energy absorption components, as well as signal acquisition and processing systems. A schematic diagram of the Hopkinson pressure bar apparatus is presented in Fig 2.

In Fig 2, the striker is cylindrical in shape, with a length of 0.4 m. It is propelled by high-pressure nitrogen gas at a pressure of 0.1MPa, achieving impact velocities in the range of 3–7 m/s. A waveform shaper, made of rubber, is positioned at the front end of the incident bar; it has a diameter of 10 mm and a thickness of 0.8 mm. The pressure bar system consists of both incident and transmission bars, which are made of aluminium. Each bar is 2.5 m in length, with a longitudinal wave velocity of 5050 m/s and an elastic modulus of 68GPa. The energy absorption system includes an energy-absorbing bar and a damping device.

When the striker impacts the incident bar, it generates an incident stress wave that propagates along the incident bar towards the coal specimen. Within the specimen, the stress wave undergoes multiple reflections and transmissions, leading to high-speed deformation and eventual failure of the sample. During this process, part of the stress wave is reflected back into the incident bar, while another portion is transmitted into the transmission bar, generating reflected and transmitted waves, respectively. Strain gauges, mounted on both the incident and transmission bars, serve as sensors for three types of stress waves. These signals are recorded and analyzed using a dynamic strain amplifier and a data processing system. The forces on both ends of the specimen remain nearly equal throughout the loading process, indicating that a state of dynamic stress equilibrium is achieved. Based on the fundamental assumption of one-dimensional stress wave theory, the dynamic tensile strength of coal can be expressed as [25]:


$$P_{\text{1}}(t)=EA(\varepsilon_{i}(t)+\varepsilon_{r}(t))$$ (1)

$$P_{\text{2}}(t)=EA\varepsilon_{t}(t)$$ (2)

$$\sigma_{t}(t)=-\frac{P_{\text{1}}(t)+P_{\text{2}}(t)}{\pi DB}$$ (3)

Where $P_{\text{1}}\left(t\right)$ and $P_{\text{2}}\left(t\right)$ are the axial forces applied to the two ends of the specimen, kN; E is the elastic modulus of the pressure bar GPa; and A is the cross-sectional area of the bar, m²; $\epsilon_{i}\left(t\right)$,${\;\epsilon }_{r}(t\backslash )$and $\epsilon_{t}(t)$ represent the incident, reflected, and transmitted strain, respectively; D and B denote the specimen’s diameter and thickness, m; and $\sigma_{t}\left(t\right)$ is the dynamic tensile strength, defined as the maximum value derived from the stress history, MPa.

## 3. Results and discussion

### 3.1. Analysis of dynamic tensile strength

**Fig 3:**
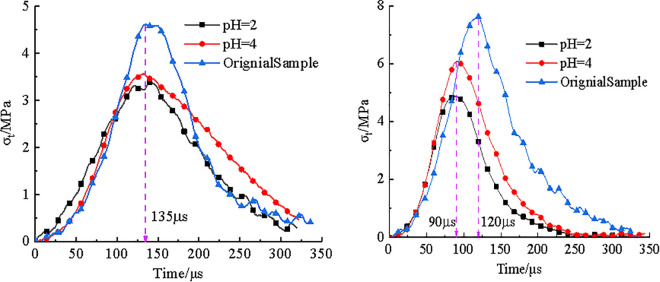
Time history curves of tensile stress in three coal samples at different impact velocities. **(a)** V = 3.6m/s, **(b)** V = 6.7m/s.

A total of 22 valid data points were obtained from the dynamic impact tests. Among them, 8 specimens were from the raw coal samples, while 7 specimens each were from coal samples treated with PH4 and PH2 acidic solutions, respectively. Fig 3 presented time-history curves of dynamic tensile strength for three types of coal specimens under different impact velocities.

As illustrated in Fig 3, the dynamic tensile strength of coal specimens decreases following exposure to acidic solutions, in order: raw coal > pH4 > pH2. In Fig 3(a), under an impact velocity of 3.6m/s, all three types of specimens failed at 135μs during dynamic loading. The dynamic tensile strength of untreated coal sample was 4.6MPa, while the strengths of specimens treated with pH4 and pH2 solutions were 3.6MPa and 3.4MPa, corresponding to reductions of 22% and 26%, respectively. In Fig 3(b), at an impact velocity of 6.7 m/s, the failure time for the raw specimen decreased to 120μs, with a 11% reduction, while the acid-treated specimens failed at 90μs, representing a 33% reduction. The dynamic tensile strength of raw sample reached 7.7MPa, while the pH4 and pH2 samples showed values of 6.1MPa and 4.9MPa, indicating strength reductions of 21% and 36%, respectively.

**Fig 4:**
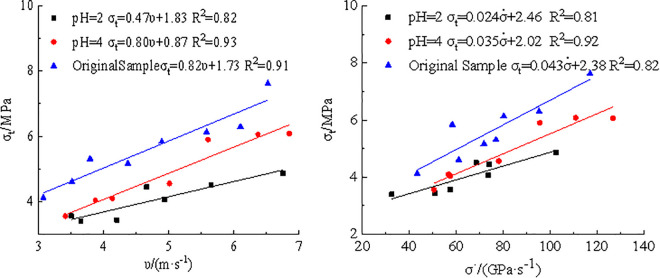
Relationship between dynamic tensile strength and impact velocity and loading rate. **(a)** Impact velocity, **(b)** Loading rate.

A rate-dependent strengthening effect was observed in dynamic Brazilian splitting tests on coal. The dynamic tensile strength exhibited a positive correlation with loading rate, reflecting the strain rate sensitivity of the material. The relationships between dynamic tensile strength, loading rate, and impact velocity for three groups of coal specimens were depicted in Fig 4.

Fig 4 showed that dynamic tensile strength of three types of coal increased approximately linearly with increasing impact velocity and loading rate. Under identical impact velocities or loading conditions, raw coal specimens consistently exhibited the highest tensile strength, followed by the pH4 samples, with the pH2 specimens showing the lowest values. The fitting coefficients further revealed that the strength increase rate of the untreated coal samples with respect to impact velocity and loading rate was the highest, followed by the pH4 group, with the pH2 group displaying the least rate of increase. These findings indicated that acid corrosion impaired dynamic strength compensation mechanism of coal, and that the greater the acidity, the more pronounced the weakening effect.

### 3.2. Energy dissipation analysis

**Fig 5:**
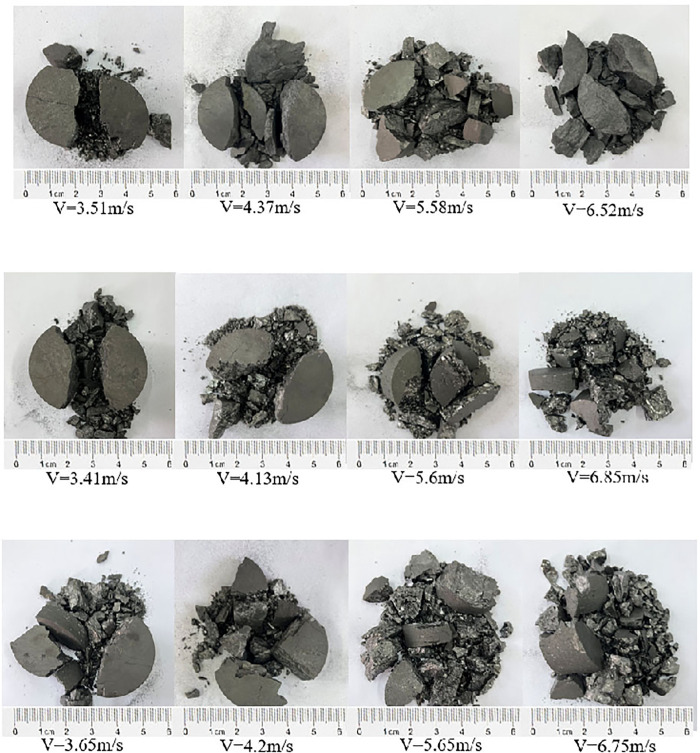
Impact failure pattern of coal samples at different impact velocities. **(a)** Impact failure pattern of raw coal sample, **(b)** Impact failure pattern of pH4 coal sample, **(c)** Impact failure pattern of pH2 coal sample.

Fig 5 presented the failure patterns of three types of coal specimens subjected to different impact velocities. As shown in Fig 5, the fragmentation characteristics of the specimens exhibited a distinct and regular variation with changing impact velocity. Both the impact velocity and the action of acidic solution significantly influenced the failure characteristics of coal samples. At lower impact velocities, coal specimens tended to fragment into larger blocks, and the dominant failure mode was tensile splitting. The main fracture typically propagated along the loading axis and penetrated the specimen entirely. Due to local compressive–shear effect at the specimen–bar interfaces during the Brazilian test, a small amount of fine fragments and powder were observed near the contact ends of the specimens [7]. As the impact velocity increased, the coal was received higher amounts of kinetic energy, leading to the development of multiple secondary cracks in addition to the main fracture. Consequently, the number of small fragments increased, the overall degree of fragmentation intensified, and the failure morphology became increasingly complex. A comparison of the fragmentation characteristics of three types of specimens under similar impact velocities revealed that acid-treated coal samples exhibited a higher degree of pulverisation, with more fine particles and small-sized fragments than the untreated samples. Moreover, the extent of fragmentation was positively correlated with the acidity of the solution—the stronger the acidity, the greater the fragmentation observed.

This behaviour can be attributed to the degradation effect induced by acidic corrosion, which led to the proliferation of internal defects and microcracks within the coal structure. As a result, the load-bearing capacity was reduced and the resistance to crack initiation diminished. Under dynamic loading conditions, more pre-existing microcracks were activated during the fracture process, while the formation of new cracks intensified. Therefore, acid-corroded specimens experienced more severe fragmentation and exhibited more complex failure morphologies.

The fragmentation of coal specimens under dynamic loading was essentially an energy dissipation process. The dissipated energy under dynamic loading indirectly reflected the difficulty of crushing test coal samples [26].

In Split Hopkinson Pressure Bar (SHPB) tests, the energy carried by stress waves can be calculated using the following equations [27]:


$$W_{I}\;=\;AEC\int_{\text{0}}^{t}\epsilon_{I}^{\text{2}}(t)dt$$ (4)

$$W_{R}\;=\;AEC\int_{\text{0}}^{t}\epsilon_{R}^{\text{2}}(t)dt$$ (5)

$${\;W}_{T}\;=\;AEC\int_{\text{0}}^{t}\epsilon_{T}^{\text{2}}(t)dt$$ (6)

Where $W_{I}$, $W_{R}$, and $W_{T}$ denote the incident energy, reflected energy, and transmitted energy respectively, J; and C represents the elastic wave velocity of the pressure bar, m·s^-1^.

Energy dissipation during fragmentation of test coal samples, $W_{FD}$, is given by:


$$W_{FD}\;=\;W_{I}-W_{R}-W_{T}$$ (7)

Based on equations (4) to (7), the incident energy, reflected energy, transmitted energy, and energy dissipation rate of three coal samples at different impact velocities were calculated. The results were summarized in Table 1.

**Table 1:** Energy distribution of coal samples.

Coal Sample Type	Impact Velocity /m·s^-1^	Incident Energy/J	Reflected Energy/J	Transmitted Energy/J	Dissipated Energy/J	Energy Dissipation Rate
Raw coal sample	3.07	9.37	6.54	0.23	2.6	0.28
	3.51	11.03	8.32	0.25	2.48	0.23
	3.79	12.58	9.09	0.39	3.1	0.25
	4.37	17.69	13.60	0.41	3.68	0.23
	4.89	22.39	15.78	0.44	6.18	0.28
	5.58	28.51	20.64	0.55	6.84	0.24
	6.1	34.02	26.21	0.44	7.35	0.22
	6.52	37.49	27.61	0.59	9.29	0.25
pH4 coal sample	3.41	11.35	7.88	0.21	3.08	0.27
	3.87	14.45	10.25	0.17	4.02	0.28
	4.13	15.17	12.23	0.44	2.51	0.18
	5.01	22.73	19.89	0.25	2.59	0.11
	5.6	27.77	23.06	0.35	4.36	0.16
	6.37	36.76	31.99	0.31	4.46	0.12
	6.85	42.03	36.59	0.33	5.11	0.12
pH2 coal sample	3.5	10.53	9.04	0.14	1.36	0.13
	3.65	14.26	11.45	0.19	2.62	0.18
	4.2	16.56	15.03	0.16	1.36	0.08
	4.66	19.47	17.43	0.22	1.83	0.09
	4.93	21.83	19.94	0.15	1.7	0.08
	5.65	29.89	25.83	0.25	3.81	0.13
	6.75	41.18	37.43	0.21	3.54	0.09

**Fig 6:**
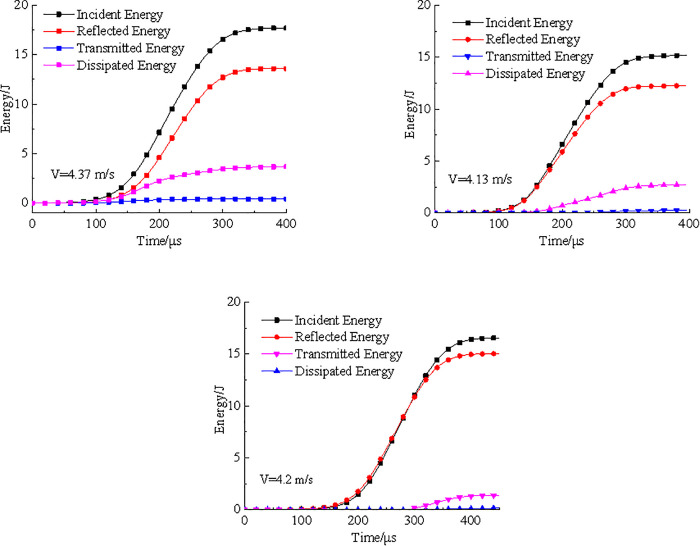
Energy time curve. (a) raw coal sample (b) pH4 coal sample (c) pH2 coal sample.

Fig 6 illustrated the time-dependent curves of incident energy, reflected energy, transmitted energy, and dissipated energy for three types of coal samples under dynamic impact loading. As shown in Fig 6, the energy evolution trends of three types of coal samples were generally similar. During the initial loading stage, both incident and reflected energies increased gradually, followed by an approximately linear growth with time. In contrast, the transmitted energy remained consistently low throughout the process, showing minimal variation. At 350μs, all energy values approached their respective peaks and subsequently tended to stabilize. Throughout the loading process, the transmitted energy remained minimal and nearly constant. This was attributed to the small contact area between the specimen and the bar in dynamic Brazilian test. When the incident energy reached the specimen, a significant portion was reflected, forming reflected energy, while most of the transmitted energy was absorbed by the specimen, with only a small fraction reaching the transmission bar.

Notably, with increasing acidity of the treated solution, the rate of increase in reflected energy became more pronounced. For the pH2 coal sample, the reflected energy in the initial loading stage exceeded the incident energy, resulting in a negative dissipated energy value. This phenomenon arose from the mismatch in acoustic impedance between the specimen and the bar, which governed the propagation of stress wave. A lower acoustic impedance in the specimen led to poorer impedance matching with the bar, thereby increasing both the magnitude and rate of reflected wave generation. Under the action of acid solution, partial dissolution of minerals into the acidic solution increased the number of pores and microcracks within the coal. These structural defects reduced the longitudinal wave velocity [28], thereby decreasing the acoustic impedance of the specimen. The more severe the acid corrosion, the greater the reduction in impedance. Compared with the untreated coal sample, the acid-treated specimens generated more reflected waves, which explained the initially higher reflected energy observed in the pH2 sample.

## 4. Numerical simulation for Dynamic Brazilian Splitting Analysis of Coal Samples

### 4.1. SHPB model

**Fig 7:**
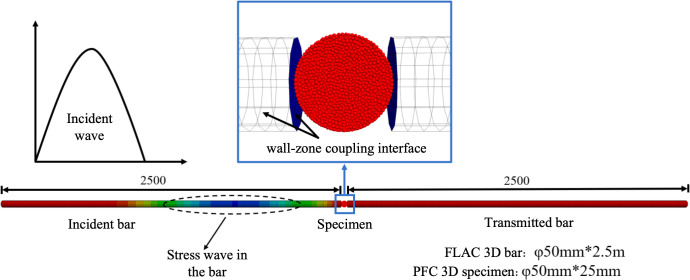
SHPB numerical model.

To further explore the dynamic tensile failure mechanism of coal under impact loading, a three-dimensional numerical model of SHPB test was established using a coupled continuum–discrete approach. Fig 7 showed the schematic diagram of SHPB model. In this model, both the incident and transmission bars are 2.5m in length, with Poisson ratio of 0.33 and a Young’s modulus of 68GPa. Monitoring points are placed at the midpoints of the incident and transmission bars to capture the propagation of stress waves through the process. The three-dimensional coal sample model, with dimensions of 50 mm*25 mm, was constructed using PFC3D software. It consists of 10,772 spherical particles, with radii uniformly distributed between 0.7 mm and 1.05 mm. Bonded contacts were assigned between adjacent particles, where the bonding gap was set to be less than or equal to 0.2 times the average particle diameter. A “wall-zone” coupling interface was applied between the bar and coal sample to enable information exchange between the discrete particles and the continuous bars.

### 4.2. Model parameter calibration

**Fig 8:**
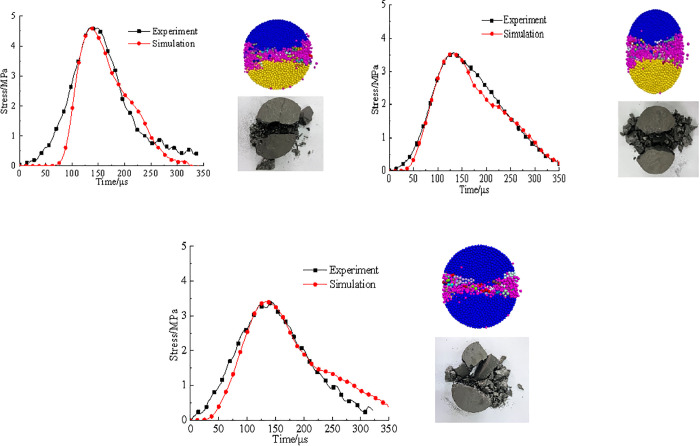
Comparison of PFC simulation and experimental results. (a) raw coal sample, (b) pH4 coal sample, (c) pH2 coal sample.

Fig 8 compared the tensile stress–time curves and failure patterns from both laboratory experiments and numerical simulations of three types of coal samples under an impact velocity of 3.6 m/s. It can be seen that the tensile stress–time curves obtained using Flat Joint Model (FJM) show good agreement with the experimental results. In terms of failure patterns, all coal samples developed a primary fracture surface along the loading axis. Due to the local compression-shear effect at the interface between coal sample and the loading bar during dynamic Brazilian splitting test, triangular crushed zones formed at both ends of the model. Overall, the failure was dominated by tensile cracking with some localized shearing, closely resembling the experimentally observed failure modes. This confirms the validity of the calibrated parameters used in the simulation.

The calibrated micromechanical parameters were adopted in subsequent simulation analyses. Table 2 presented the detailed parameters used in the FJM model. It is worth noting that the duration from initial loading to peak stress in the FJM-generated tensile stress curve is shorter than that observed in the laboratory test. This discrepancy is primarily due to the use of rigid particles in PFC, which do not accurately capture the compaction behaviour that occurs in natural coal samples under dynamic loading.

**Table 2:** Micro-parameters of the FJM model.

Micromechanical Parameter	Raw Coal Sample	pH4 Coal Sample	pH2 Coal Sample
Bond installation gap $g_{c}$(mm)	0.28	0.28	0.28
Number of radial and circumferential elements Nr, Nα	1,3	1,3	1,3
Effective modulus of particles and bonds (GPa)	8.5	3.2	2.45
Stiffness ratio	2.5	2.5	2.5
Cohesive strength (MPa)	30.35	21.31	18.12
Normal bond strength (MPa)	6.05	4.35	3.6
Friction angle (°)	20	20	20
Friction coefficient	0.3	0.3	0.3

### 4.3. Analysis of dynamic simulation results

#### 4.3.1. Dynamic mechanical response.

**Fig 9:**
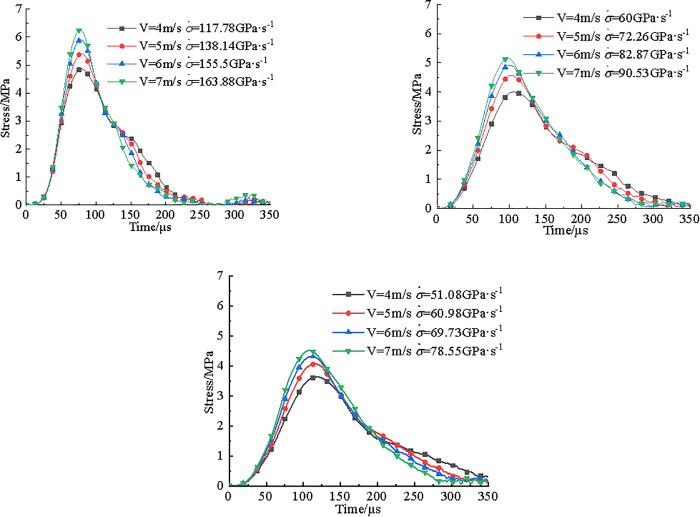
Tensile stress time history curves of coal sample. (a) raw coal sample, (b) pH4 coal sample (c) pH2 coal sample.

Fig 9 presented the tensile stress-time curves of FJM models for three types of coal samples under different impact velocities. As shown in Fig 9, during dynamic loading, the models exhibited a short compaction phase, followed by a linear increase in tensile stress with time. Once the peak was reached, the stress dropped rapidly, indicating a pronounced brittle failure behavior. Under impact loading, FJM models also exhibited a clear strain rate effect. With increasing impact velocity, both loading rate and dynamic tensile strength of three FJM models of coal samples increased accordingly.

**Fig 10:**
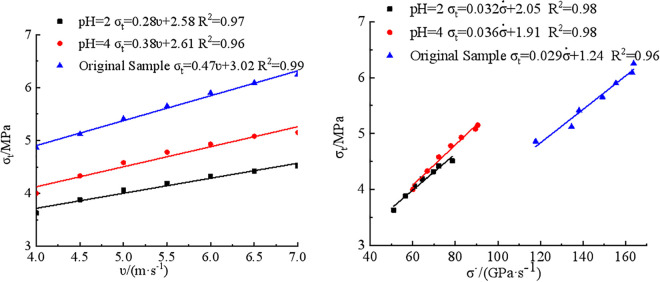
Relationship between dynamic tensile strength and impact velocity and loading rate. (a) impact velocity (b) loading rate.

Fig 10 illustrated the relationship between the dynamic tensile strength of three FJM models of coal samples and the impact velocity and loading rate.

As shown in Fig 10(a), the dynamic tensile strength of three models of coal samples increased linearly with the rise in impact velocity. Under the same impact velocity, the dynamic tensile strength followed the order: raw coal model > pH4 model > pH2 model. Furthermore, the rate of increase in dynamic tensile strength with impact velocity also followed the same order: raw coal model> pH4 model> pH 2 model. From Fig 10(b), it can be observed that with increasing loading rate, the pH4 coal model exhibited the greatest increase in dynamic tensile strength, while the raw coal model showed the smallest. This trend deviated from the experimental results to some extent. The reason lied in the presence of weak discontinuities in experimental coal samples, such as bedding planes, joints, and pores. Chemical reactions between acidic solutions and the inorganic minerals and organic matter in the coal lead to alterations in mineral composition and molecular structure, resulting in more complex micro-structures.

#### 4.3.2. Damage evolution characteristics.

**Fig 11:**
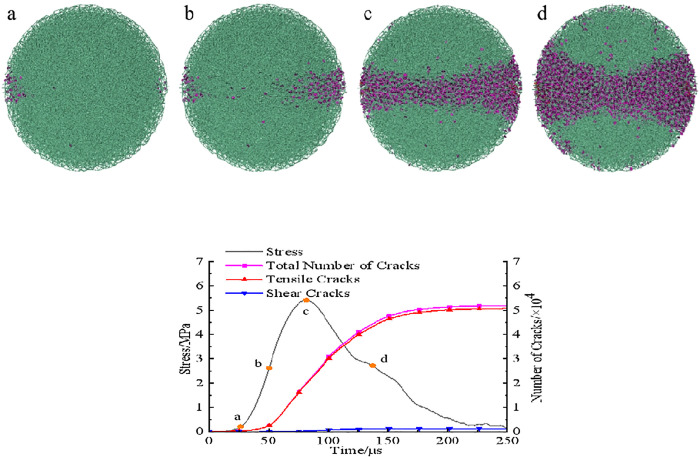
Damage evolution of raw coal model under dynamic splitting condition. **(a)** Microcrack distribution **(b)** Stress and microcracks.

In PFC, the crack behavior throughout the entire spatial domain during the failure process of coal sample can be observed in real-time, allowing direct identification of the types and quantities of microcracks and facilitating analysis of the dynamic fracture mechanism of coal sample. Taking the damage evolution process of the raw coal model at an impact velocity of 5 m/s as an example, Fig 11 illustrated the damage evolution under dynamic splitting conditions.

Fig 11(a) showed the damage and failure states at four stress points a, b, c, and d marked in

Fig 11(b), while Fig 11(b) presented the dynamic stress-time curve of the model alongside the cumulative microcrack count. From Fig 11(b), at the initial stress rising stage (point a), the model was approximately in the linear elastic deformation phase, with only a few microcracks generated. With loading more, stress concentration near the interface between the model and the bar end became increasingly pronounced, accelerating the breakage of bonds on both sides of the model. Microcracks converged to form high-density crack clusters in a wedge-shaped region on both sides of the model. Subsequently, the number of microcracks rapidly increased with the rising dynamic stress, indicating aggravated structural damage to the model. When the stress reached its peak at point c, the microcracks have merged in the middle of the model to form a through-going fracture band, and the model has completely lost its load-bearing capacity. During the unloading phase, microcrack growth continued but at a slower rate. When stress was relieved at point d, the main macroscopic crack responsible for the model failure has formed. New cracks during unloading mainly appear in the heavily damaged zones at the left and right ends of the model, with a few scattered in the lightly damaged regions on the top and bottom sides. After passing stress stage point d, the crack growth rate further decreased, and after 200μs, the number of cracks gradually stabilized.

The total number of cracks at failure was 52,342, among which tensile cracks accounted for 51,111 and shear cracks for 1,231. The vast majority of microcracks were tensile in nature, with shear cracks constituting only 2.35%, mainly scattered in the contact area between the model and the bar end.

**Fig 12:**
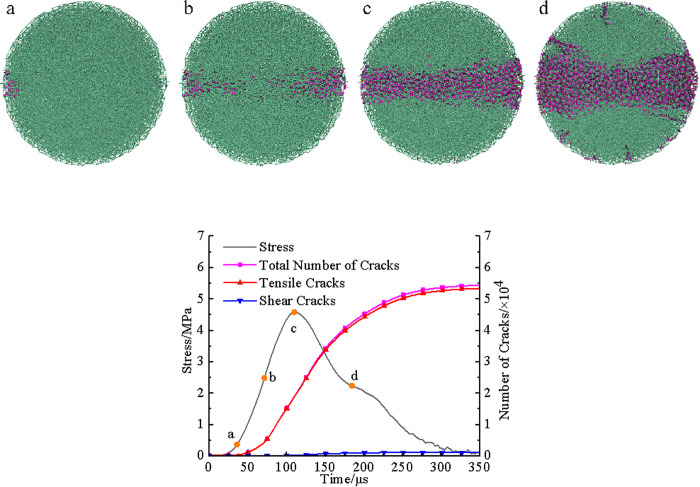
Damage evolution of the pH = 4 model under dynamic splitting condition. (a) Microcrack distribution (b) Stress and microcracks.

**Fig 13:**
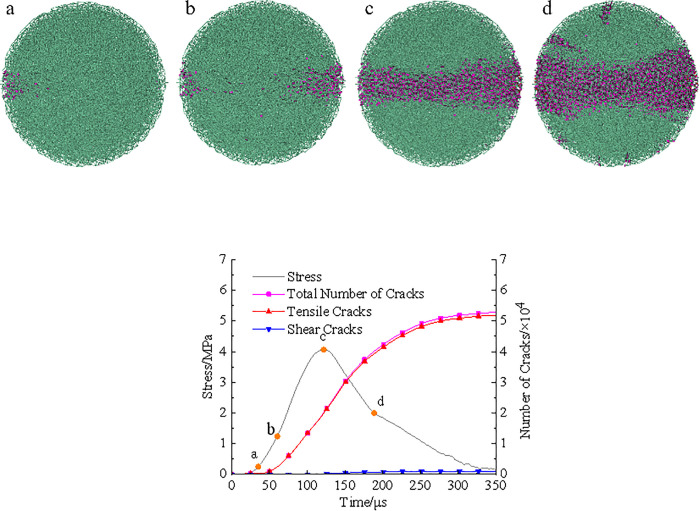
Damage evolution of the pH = 2 model under dynamic splitting condition. (a) Microcrack distribution (b) Microcrack distribution.

Figs 12 and 13 depicted the dynamic damage evolution processes of pH4 and pH2 coal sample models, respectively, with failure processes generally similar to those shown in Fig 11.

**Fig 14:**
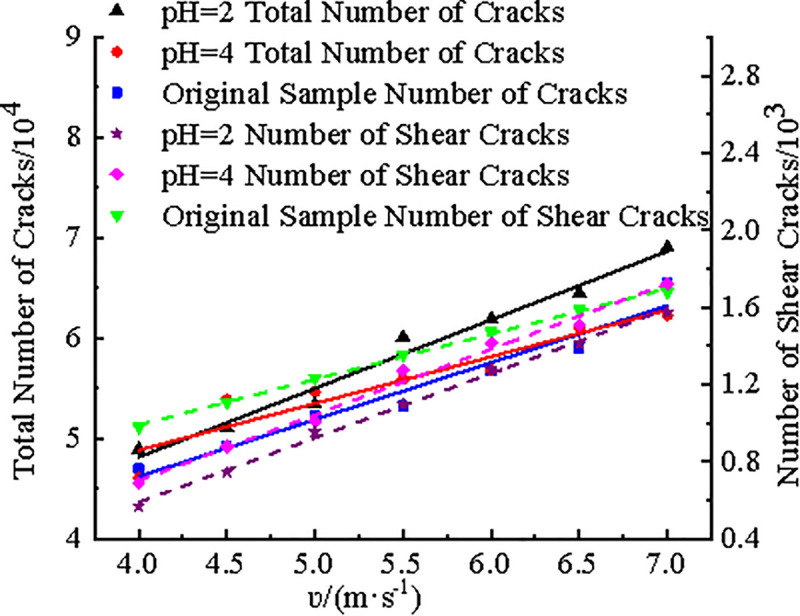
The relationship between number of microcracks and impact velocity.

Fig 14 showed the variation curves of the total number of cracks and the number of shear cracks after failure for three coal sample models with increasing impact velocity. It can be observed from Fig 14 that both the total number of cracks and the number of shear cracks increase linearly with the impact velocity for three models. Specifically, the total number of cracks for raw coal models ranged from 46,984–65,549, for the pH4 model from 46,032–62,281, and for the pH2 model from 48,884–69,054. With the same impact velocity, the total crack count ranked as pH2 model > pH4 model > raw coal model. Although the number of shear cracks also increased with impact velocity, their quantity remained relatively small, ranging between 566 and 1,676, accounting for no more than 3% of the total cracks at any given velocity. The microcrack statistics indicated that tensile cracks dominated throughout the dynamic splitting process, with tensile interactions between particles being the primary cause of model damage and failure.

#### 4.3.3. Failure mode analysis.

**Fig 15:**
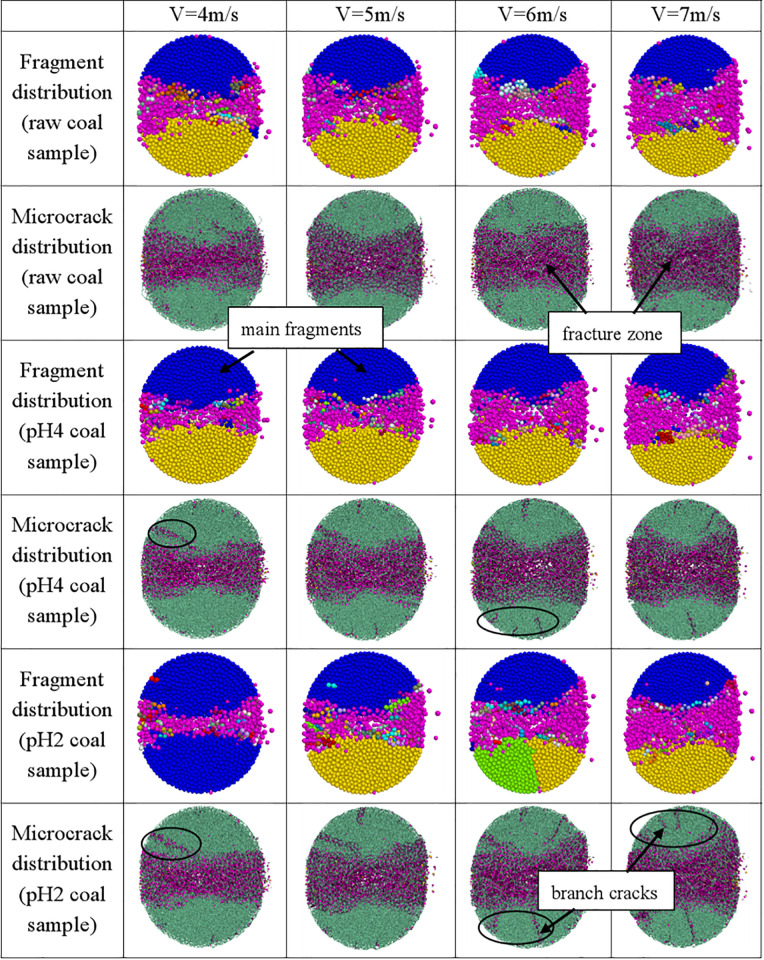
Failure pattern of three samples at different impact velocities.

Fig 15 summarized the distribution characteristics of fragments and microcracks after dynamic splitting failure for three coal sample models. It can be seen from Fig 15 that the fragment distribution features of three models were generally similar. Initially, microcracks mainly developed at both ends of the model and gradually propagated and coalesced toward the center, eventually forming a through-going fracture band. Along the loading axis, the model was split into two main fragments, upper and lower parts. In the end regions of the model, due to stress concentration effects, the inter-particle adhesive bonds in this area were severely damaged, with many small fragments peeling off and particles flying away, forming a triangular damage zone. The model exhibited an X-shaped failure pattern characterized by tensile cracking accompanied by localized shear failure. With increasing impact velocity, the sizes of the main fragments decreased gradually, and the distance between two main fragments increased, while the number of small fragments and particles flying away gradually increased, indicating progressively more severe damage to the model.

The microcrack distribution characteristics of three coal sample models differed notably. For the raw coal model, a damaged crack zone can be observed at the center of the model. In addition, some scattered adhesive bond damage appeared on the main fragments. The damage area gradually expanded with increasing impact velocity, but the number of adhesive bond damages on the main fragments remained relatively small, and no continuous long cracks were formed. In contrast, at an impact velocity of 4 m/s, the pH4 model exhibited a greater number of adhesive bond damage on the main fragments, with the emergence of an oblique long branch crack. As the impact velocity increased, the number of branch cracks on both main fragments also rose. The pH2 model showed a similar trend; at an impact velocity of 6 m/s, the branch cracks on the lower main fragment fully penetrated, resulting in three large fragments. Under the same impact velocity, the pH2 model has the highest number of branch cracks on the main fragments, with the most complex crack distribution. The fracture patterns predicted by the numerical simulation closely resembled the evolution characteristics observed in the dynamic tests.

## 5. Conclusions

Dynamic Brazilian splitting tests were conducted using a SHPB apparatus on raw coal samples and those treated with acidic solutions of pH4 and pH2. The dynamic tensile strength and energy dissipation characteristics of the samples were analyzed. A three dimensional SHPB numerical model was established to simulate the dynamic splitting process, and the simulation results were further analyzed in terms of dynamic mechanical response, damage evolution, and failure modes. The main conclusions were as follows:

(1) The action of acidic solutions significantly weakened the dynamic tensile strength of coal. Compared to raw coal samples, the dynamic tensile strength of the pH4 and pH2 treated samples decreased by approximately 22% and 36%, respectively. The failure time of coal was inversely correlated with the impact velocity; under high-speed loading, acid-corroded coal failed more rapidly. The dynamic tensile strength showed a clear rate dependency, increasing with the loading rate.
(2) Under dynamic loading, the tensile strength of coal, fragmentation degree, and energy dissipation increased with the loading rate. Acidic treatment reduced both the dissipation energy and its growth rate. The stronger the acidity, the more violently the chemical reaction between mineral particles and the matrix in coal with cations in the solution, producing dissolution pores and microcracks, leading to a deterioration in the flatness and cohesion of the coal matrix [29], the lower the energy dissipated during coal fragmentation.
(3) The numerical simulations successfully reproduced the dynamic fracture process of coal and enabled quantitative analysis of micro-scale cracking. The number of cracks and the degree of fragmentation increase with impact velocity. Under the same impact conditions, models exposed to stronger acid solutions exhibited more complex crack patterns and a higher degree of fragmentation, consistent with the experimental observations.
(4) The final failure mode of coal involved a combination of microscopic tensile cracking and macroscopic tensile-shear failure. During dynamic loading, tensile stress concentrated in the centre of the specimen and primarily governed the propagation path of the main cracks, while compressive stress localized at both ends of the specimen, causing shear damage in those regions. High localized compressive stress is the fundamental cause of end-region shear failure.
